# Generation of chimeric kidneys using progenitor cell replacement: Oshima Award Address 2021

**DOI:** 10.1007/s10157-022-02191-3

**Published:** 2022-02-09

**Authors:** Shuichiro Yamanaka

**Affiliations:** Division of Nephrology and Hypertension, Department of Internal Medicine, The Jikei University School of Medicine, Tokyo, Japan

**Keywords:** Progenitor, Chimera, Hybrid, Regeneration, Induced pluripotent stem cells, Development

## Abstract

It is believed that the development of new renal replacement therapy (RRT) will increase treatment options for end-stage kidney disease and help reduce the mismatch between supply and demand. Technological advancement in the development of kidney organoids derived from pluripotent stem cells and xenotransplantation using porcine kidneys has been accelerated by a convergence of technological innovations, including the discovery of induced pluripotent stem cells and genome editing, and improvement of analysis techniques such as single-cell ribonucleic acid sequencing. Given the difficulty associated with kidney regeneration, hybrid kidneys are studied as an innovative approach that involves the use of stem cells to generate kidneys, with animal fetal kidneys used as a scaffold. Hybrid kidney technology entails the application of local chimerism for the generation of chimeric kidneys from exogenous renal progenitor cells by borrowing complex nephrogenesis programs from the developmental environment of heterologous animals. Hybrid kidneys can also utilize the urinary tract and bladder tissue of animal fetuses for urine excretion. Generating nephrons from syngeneic stem cells to increase self-cell ratio in xeno-tissues can reduce the risk of xeno-rejection. We showed that nephrons can be generated by ablation of host nephron progenitor cells (NPCs) in the nephron development region of animals and replacing them with exogenous NPCs. This progenitor cell replacement is the basis of hybrid kidney regeneration from progenitor cells using chimera technology. The goal of xeno-regenerative medicine using hybrid kidneys is to overcome serious organ shortage.

## Introduction

Due to aging of the population and the prevalence of lifestyle-related diseases such as diabetes and hypertension, the number of patients with renal failure is increasing rapidly. It is estimated that more than 5 million people will require renal replacement therapy (RRT) by 2030. It was reported that, in 2010, more than 2 million patients died prematurely due to the unavailability of RRT, and shortage of RRT is a global concern [1]. Renal transplantation is the most effective RRT [2]; however, supply of donor organs is limited, and it is difficult to cope with the rapidly increasing number of patients when there is an organ shortage. If induced pluripotent stem (iPS) cells, which have unlimited proliferative capacity, can be used as a cell source for kidney regeneration, organ regeneration can overcome the limitations of transplant organ donation and significantly improve the outcomes of patients with end-stage kidney disease (ESKD). However, the kidney performs many functions regarding homeostasis, and due to its complex three-dimensional (3D) structure, the kidney is difficult to regenerate. To overcome this difficulty, studies have been conducted to generate whole kidneys using different animal developmental environments. Results have started to emerge in recent years, and in this paper, I present these results and report my own research findings.

## Approaches to kidney regeneration

**Fig. 1:**
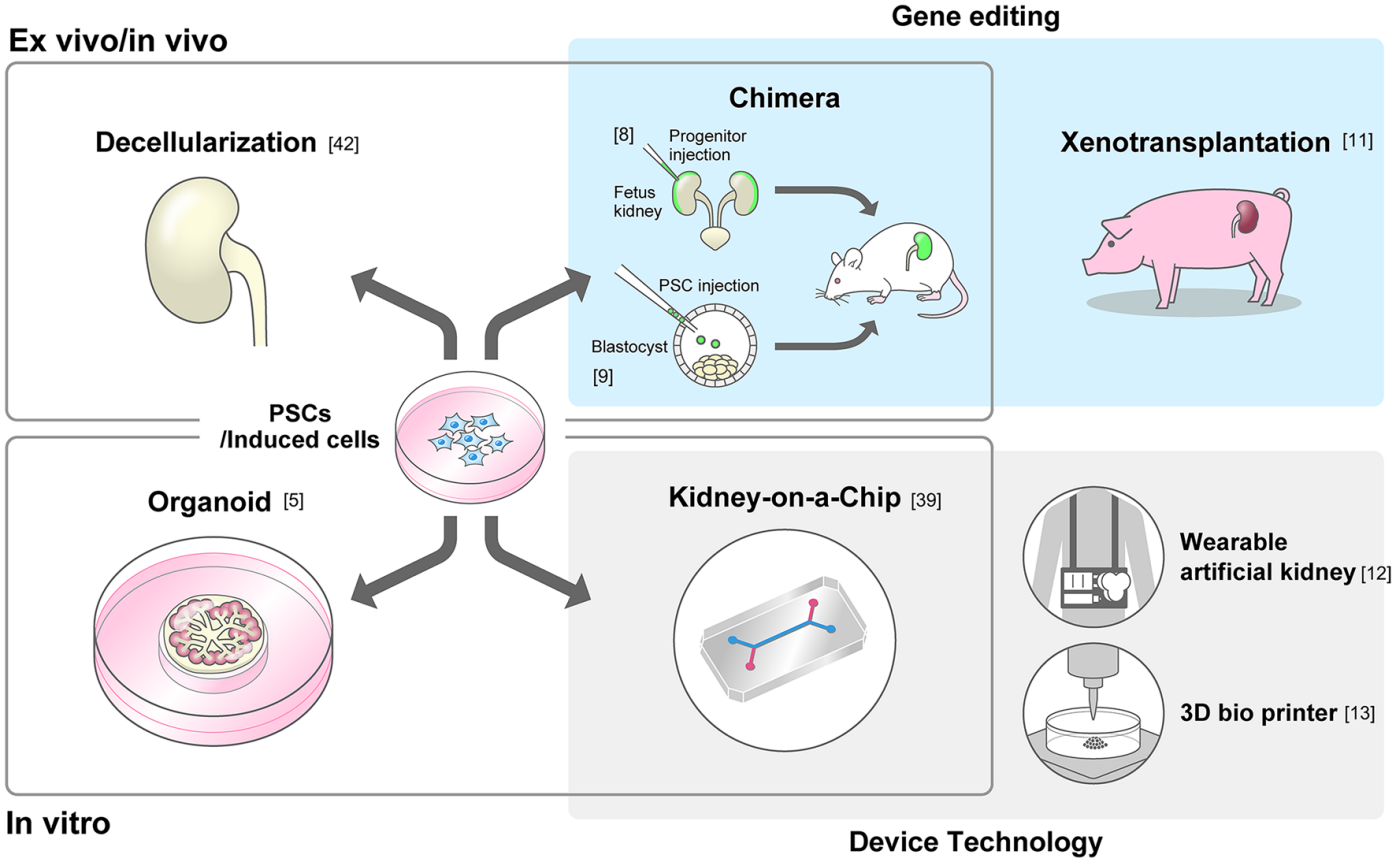
Current technology of kidney generation. The technology to generate kidneys in vivo is presented in the upper section. Decellularization uses a scaffold in which cells are removed using a detergent, leaving only the extracellular matrix. Chimera technology has two strategies: blastocyst complementation using PSCs as donor cells and embryonic organ complementation using progenitor cells. In both chimera and xenotransplantation technologies, genome editing technology is applied to the heterologous tissue parts. Xenotransplantation does not utilize stem cells. The bottom row shows the technology for in vitro kidney generation. Kidney organoids derived from PSCs have been the most widely studied in recent years. The scaffold is a kidney on a chip fabricated using Device technology based on artificial substrate. There are also engineering technologies, such as wearable artificial kidneys and 3D bioprinters that aim to develop renal replacement therapy. *PSCs* pluripotent stem cells

The use of iPS cells for kidney regeneration is ethical because it does not involve destruction of embryonic cells. Furthermore, with regard to immune rejection, the availability of autologous stem cells for organ regeneration is of great significance. Regenerative research has been accelerated by the discovery of iPS cells (Fig. 1, Table1) [3]. In addition, improvements in comprehensive analysis techniques such as single-cell ribonucleic acid sequencing have allowed for detailed analysis of renal development, and significant progress has been made in differentiation induction techniques that mimic the developmental process from pluripotent stem cells to cells of kidney lineage. Organoid technology, which combines multiple cell types and utilizes their self-organizing ability to create tissue-like cell populations, is aimed at in vitro organ regeneration only, and it is the mainstream of current kidney regeneration methods [4, 5]. Decellularization technology, which uses organs from which cells have been removed as a scaffold for cell differentiation, and kidney-on-a-chip technology, which uses artificial chips on which cells are seeded, are also technologies that were greatly influenced by the discovery of iPS cells (Fig. 1) [6, 7]. In previous studies, a hybrid organ regeneration method that uses chimeric technology to generate organs from exogenous stem cells by borrowing the developmental environment of animals was reported [8–10]. Studies are ongoing in the area of xenotransplantation, which does not rely on pluripotent stem cells and uses gene editing technology to make heterologous animal organs less immunogenic under the control of multiple immunosuppressive agents [11]. Wearable artificial kidneys and 3D printers based on engineering technology are also being studied [12, 13]. Of these technologies, chimeric technology, which has the capability for blood filtration and endocrine secretion in vivo, is promising for the regeneration of 3D organs.

**Table 1:** Comparison of technical issues in kidney regeneration technologies

				Chimera technology		Xenotransplantation
	Kidney-on-a-chip	Organoid	Decellularization	Fetus organ complementation	Blastocyst complementation	
Vascular structure	Not included (or rebuild) [38]	Not included (or rebuild) [7, 39]	Included (rebuild/native) [40, 41]	Included (rebuild/native) [7, 27]	Included (native) [8, 28]	Included (native) [10]
Urinary tract	Not included	Not included	Included	Included [7, 20, 27]	Included [8, 28]	Included [10]
Off target problem*	N/A	No****	Unknown	No (under review)	Concern	N/A
Immunosuppressive regulation**	N/A	No immunosuppression***	Low–moderate*** [42]	Moderate (under review)	Moderate (assumption)	Severe
Main using field	In vitro	In vitro	In vivo	In vivo	In vivo	In vivo
Types of cells used*****	PTECs [43]	PSCs [43]	PSCs/differentiated cells [44]	Progenitor cells [7, 20, 27]	PSCs [8, 28]	N/A
Application of iPS technology	+ [43]	+ [36]	+ [44]	+ [21]	+ [8, 28]	N/A
Application of gene editing technology		Applicable	Applicable [42]	Essential technology	Essential technology	Essential technology
Advantages	Recapitulate mechanical strain and fluid shear stress. [45]	Composed entirely of autologous stem cells	ECM as a scaffold has bioactive substrate	The urinary tract is available and the vascular network is of recipient origin	Functional vascular network and urinary tract available	Functional vascular network and urinary tract available
	Culture time is faster [45]	High throughput experiments are possible	Native 3D structure is utilized as a scaffold	Autologous stem cell-derived parts have the potential to reduce rejection	Autologous stem cell-derived parts have the potential to reduce rejection	Existing surgical techniques for transplantation can be used
Disadvantages	Mostly PTECs seeded model [45]	No urinary tract; long-term maturation is hard	Recellularization is difficult in complex organs	Lack of validation in large animals	Donor cell contamination issues in the nervous system and germ line	Requires severe immunosuppressive control
	Difficult to generate a variety of cells [45]	Difficulty in increasing the size owing to a poor vascular network	A large number of cells are needed to seed an entire organ	The problem of distal interspecies barriers	The problem of distal interspecies barriers	There are concerns of zoo noses under severe immunosuppression

*N/A* not applicable; *PSCs* pluripotent stem cells; *PTECs* proximal tubular epithelial cells; *ECM* Extracellular matrix

*The off-target problem is contamination of recipient's central nervous system or germ line with the cell source

**Possible rejection and immunosuppressant control during clinical application

***The immune response differs depending on whether the donor kidney is of human or animal origin

****10–20% of the cells differentiated into cells other than kidney; however, contamination of the central nervous system or germ cells has not been evaluated

*****About the cell source used as donor cells

## Three renal progenitor cells that play a leading role in kidney development

**Fig. 2:**
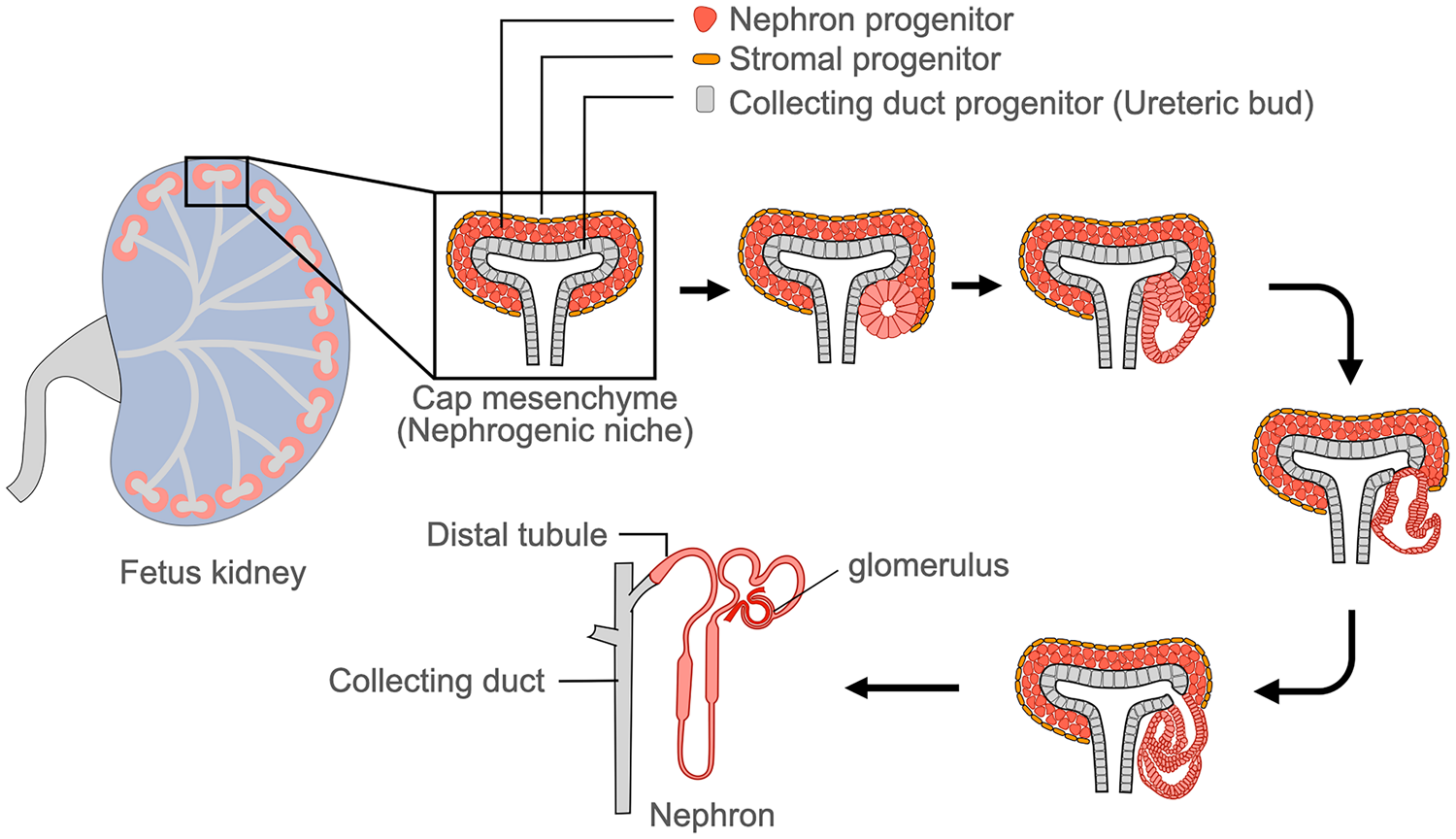
Nephrogenic niche composed of renal progenitor cells and nephron development. The starting point for nephron development is located under the renal capsule of fetal kidneys. This nephrogenic niche is composed of a nephron progenitor, stromal progenitor, and collecting duct progenitor (ureteric bud), which generate nephrons

The kidney is composed of three major types of renal progenitor cells: nephron progenitor cells (NPCs), ureteric buds (UBs), and stromal progenitor cells (SPCs). Glomeruli, proximal tubules, and distal tubules differentiate from NPCs, while the collecting duct (CD) and part of the ureter differentiate from UBs, which are CD progenitor cells. Mesangial cells, pericytes, renin-producing cells, and stromal cells differentiate from SPCs. UBs have a bud-like morphology and are surrounded by NPCs and SPCs. Cap mesenchyme (CM), which is the origin of nephron development and contains NPCs and SPCs, forms a nephrogenic niche with the tip of UB [14]. Progenitor cells are capable of self-renewal, and the nephrogenic niche increases in number with advancing fetal age, simultaneously producing nephrons (Fig. 2). NPCs committed to nephron differentiation are balanced by self-renewal/population expansion NPCs, which are regulated by signals from surrounding SPCs [15]. In brief, NPCs, SPCs, and UBs are the three essential progenitors of normal nephrons.

## The nephrogenic niche easily takes up exogenous cells

Exogenous NPCs can be implanted and integrated into CM [8, 16]. Just under the capsule of the fetal kidney, there is a paved area of nephrogenic niches known as the nephrogenic zone. Injected cells spread into the space between the renal capsule and the nephrogenic niche; this was demonstrated by inserting a glass tube through the renal pelvis of a fetal kidney and stopping when the tip was just below the membrane to expel the cells in the glass tube without disrupting the renal capsule [8]. This method is termed PAra Nephrogenic zone Direct Approach (PANDA), and fluorescently labeled allogeneic NPCs were transplanted using PANDA, which showed the integrated transplanted cells in the native CM [17]. Cells transplanted into the nephrogenic zone were able to attach to the neighboring CM. In addition, exogenous NPCs established in the CM after transplantation were able to differentiate into nephrons together with host NPCs. In addition to nephron regeneration by transplantation of NPCs, mesangium can be regenerated by transplantation of sorted selective SPCs [18]. However, in the regeneration of nephrons and mesangium, host cells and donor-transplanted cells were mixed to form CM, and the differentiated nephrons contained both cell types in a mosaic state. The transplantation of wild-type fetal kidneys with PANDA-delivered exogenous progenitor cells to the CM shows that nephrons can be generated from exogenous renal progenitor cells by contributing to the nephrogenic niche.

## Generation of high-purity nephrons by progenitor cell replacement

**Fig. 3:**
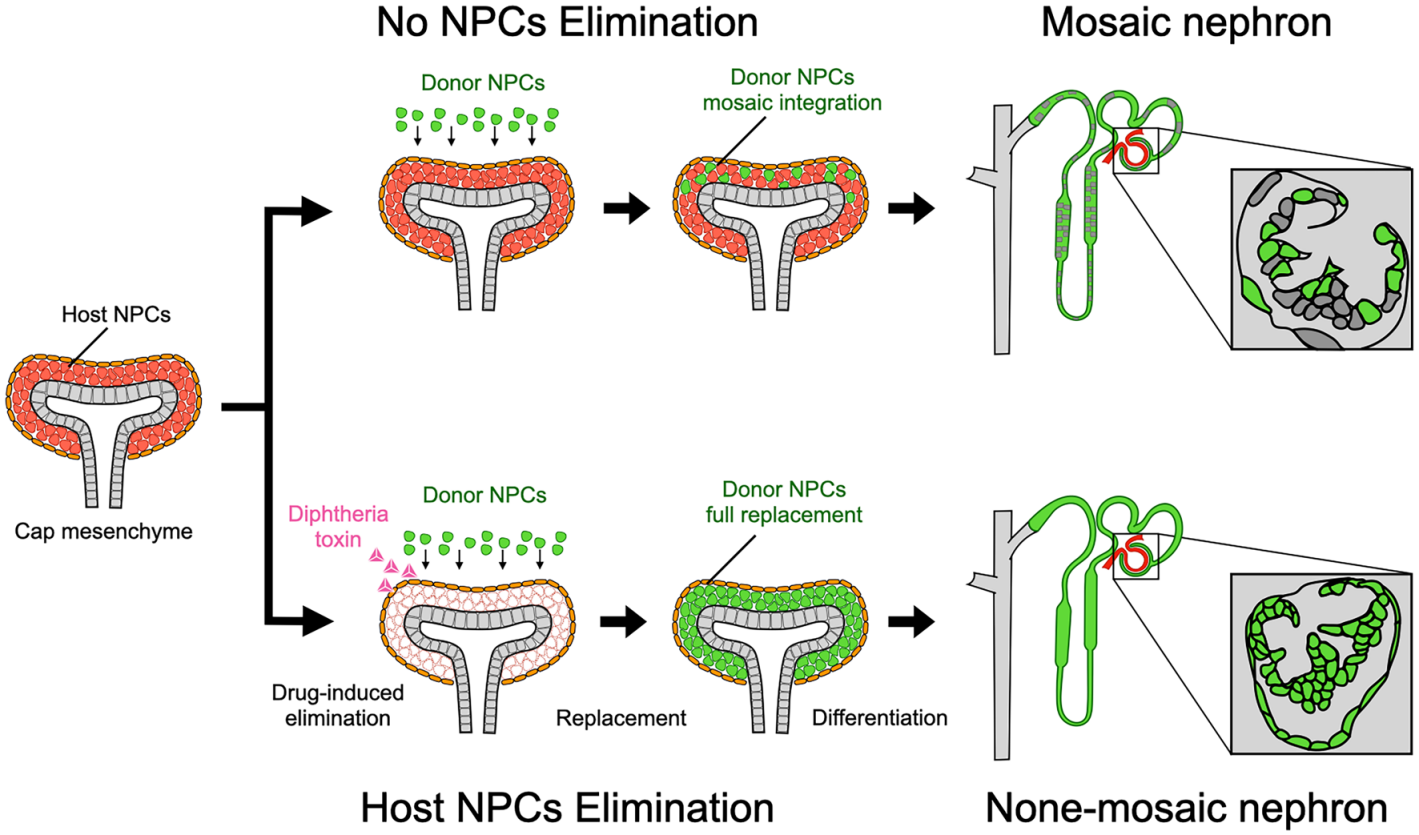
Progenitor cell replacement and nephron regeneration by inducible ablation of nephron progenitor cells. When an exogenous NPC is transplanted into a nephrogenic niche, it commits to the niche and generates a mosaic nephron consisting of host and donor NPCs. When host NPCs are removed using the diphtheria toxin (removal agent), only donor NPCs occupy the niche, and a non-mosaic, highly pure nephron is generated. This phenomenon is termed progenitor cell replacement. *NPCs* nephron progenitor cells

Nephrons generated by cell delivery to CM are a mosaic of host and donor cells. To generate nephrons derived entirely from transplanted cells, all host NPCs in the nephrogenic niche are removed, and exogenous NPCs are transplanted into the empty nephrogenic niche. Transplantation into a knockout model with the phenotype of renal deficiency was considered unsuitable for ablation of NPCs on the host side. The basis for this consideration is that the nephrogenic niche for accepting and differentiating NPCs would not exist if nephrons could not form at the start of development. Therefore, it would be expected that the proper developmental environment will be lost and that new nephrons will not be generated. This method is different from the blastocyst complementation method, in which the defect is complemented by transplanted pluripotent stem cells from early development. For transplantation into fetal organs, it was thought necessary to have a system that inductively removes cells only during kidney development. Therefore, focus was placed on the induced diphtheria toxin receptor (iDTR) system, which is a mouse line that expresses diphtheria toxin receptors (DTRs) only in specific cells in a tissue-specific Cre-dependent manner [19]. Since mice do not originally have DTRs, apoptosis of specific cells was selectively induced by administering diphtheria toxin (DT), and the Cre-loxP system was used to ensure DTR expression only in the specific cells. Therefore, attention shifted to Six2, a transcription factor specific to NPCs, and fetal kidneys (Six2-iDTR mice) obtained by crossing Six2-cre and loxP-DTR were used [8, 20]. When NPCs were excluded from the niche, exogenous NPCs were transplanted to replace the progenitor cells in the niche. The nephrons generated from the transplanted NPCs that attached to the CM after removal of the host progenitor cells were nephron structures composed only of cells transplanted due to absence of host cells. The generated nephrons were also connected to the host CDs, and passage of urine in vivo was confirmed [8, 21, 22]. The exchange of host and donor progenitor cells within the nephrogenic niche was termed progenitor cell replacement (Fig. 3). Progenitor cell replacement was observed even when the nephrogenic niche was of rat origin and the transplanted NPCs were of mouse origin, and a connection between the nascent nephron and the host CD was observed. These findings may become the basic technology for future chimeric kidney regeneration between large animals and humans. Furthermore, a progenitor cell induction and ablation system was devised, and it can be used for human cells with no need for DT, which is toxic to human cells [22]. Using an improved tamoxifen-driven progenitor cell replacement model that utilizes CreERT2, NPCs derived from human iPS cells were used as donor cells, and human iNPCs were allowed to integrate in the mouse niche, resulting in the formation of vesicles connected to the mouse UB tip [22]. Cell–cell adhesion was observed between distal interspecies cells of human and mouse origin, indicating the presence of some cross-species adhesion factors in the nephrons. Since the generated nephrons are still immature, it is necessary to demonstrate their long-term survival in vivo as they mature.

## Two strategies for the generation of chimeric kidneys via progenitor cell replacement

**Fig. 4:**
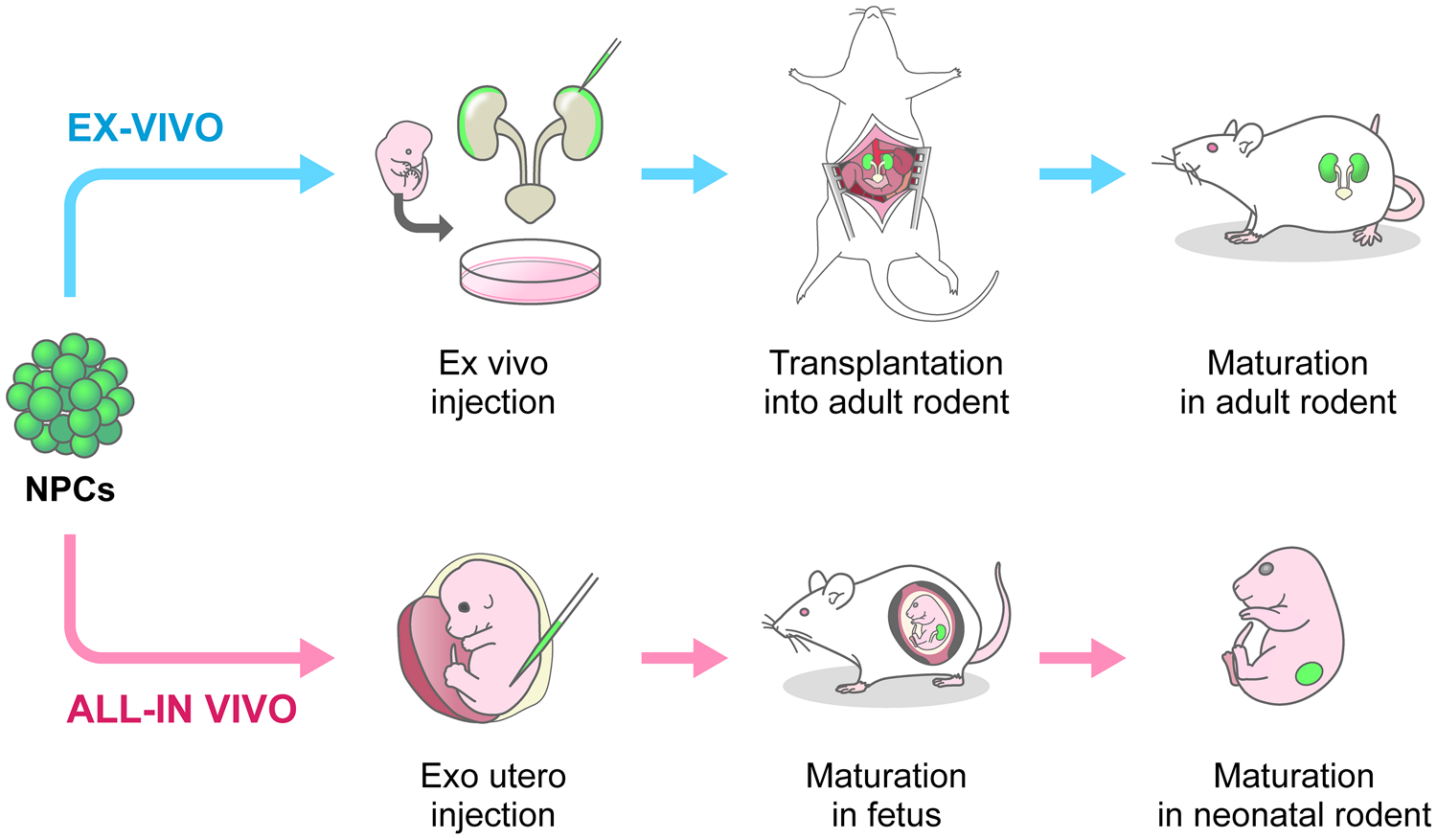
Schematic representation of transplantation method for kidney regeneration using fetal organs and local chimerism. In the upper schema, kidneys are removed from the fetus and NPCs and removal agents are injected into the nephrogenic niche on the dish. Subsequently, the fetal kidney to which the bladder and ureter was connected is transplanted into the retroperitoneum of an adult rat. NPCs are replaced and nephron differentiation proceeds in the adult rat. In the lower schema, cells are injected directly into the fetus in the mother's womb and allowed to develop in the mother while NPC replacement and nephron differentiation continue in the fetus. *NPCs* nephron progenitor cells

With delivery of progenitor cells into the nephrogenic niche and timed inducible removal of host progenitor cells, nephrons composed entirely of exogenous cells can be generated in the microenvironment of nephrogenesis. Two methods for transplanting donor cells and drugs into the fetal kidney for progenitor cell replacement were developed, namely the ex vivo method and the all-in vivo method (Fig. 4). In the ex vivo method, PANDA is used. It involves removal of a fetal kidney and the transplantation of cells and elimination agent (DT or 4OH-tamoxifen) into an extracted kidney in a petri dish. Fetal kidneys loaded with cells and elimination agent can be cultured in vitro and transplanted into the retroperitoneum of adult animals to develop in vivo (Fig. 4). Following transplantation into the recipient's retroperitoneum, blood vessels spontaneously begin to enter the transplanted kidney from the recipient's side. In previous studies, it was verified that the recipient's blood vessels enter the generating glomerulus, which filters the blood [8, 21, 22]. The advantage of the ex vivo method of transplantation is that, after transplantation, the vasculature is almost entirely of recipient origin, since the transplanted fetal kidney has no vessels. This phenomenon was previously observed in various species [23]. Since endothelial cells are the most important target of immune rejection in xenotransplantation, it is significant that blood vessels of the transplanted organ are of autologous origin and not a target of immune rejection [24]. Furthermore, in an earlier study, an improved method of fetal kidney transplantation under the retroperitoneum was developed; it involved simultaneous transplantation of the fetal kidney, ureter, and bladder without resection [25]. The fetal bladder serves as a buffer storage site for produced urine. By transplanting the entire urinary system, hydronephrosis can be prevented. Furthermore, it was confirmed that urine can be continuously drained from the recipient by connecting the host ureter to the fetal bladder, which stores urine [stepwise peristaltic ureter (SWPU) system] [25]. Fetal tissues are considered less antigenic than adult tissues [26], the vasculature is autologous, and the renal parenchyma can be replaced with syngeneic tissue, which may reduce immune rejection compared to xenotransplantation. The ex vivo hybrid kidney, which is based on fetal tissue, may be used as a workaround to reduce the intense immune rejection that has been a challenge in xenotransplantation. These experiments represent an important proof of concept for xeno-regenerative medicine [27]. However, the kidneys of the rodent interspecies chimeras developed using this technology have not demonstrated an ability to support life in the ESKD model wherein both kidneys had been extracted. A limitation of chimeric kidney regeneration technology using fetuses is that kidneys regenerated based on fetal kidneys are smaller than adult kidneys and have immature regenerating nephrons. However, we believe that it is possible to compensate for the lack of kidney function by transplanting multiple small kidneys. In terms of maturation, the SWPU system could be used to maintain the fetal kidneys in the host body for a long period of time, allowing them to mature in vivo. The SWPU system, which makes the creation of an alternative urinary tract in mice difficult, given its exceedingly small size, can be implemented in pigs, because they are larger. By inserting a progenitor cell replacement system and transplanting multiple kidneys to increase the number of kidneys in pigs, we believe that it will be possible to demonstrate life support in the ESKD model. This study is nonetheless ongoing.

The second method is to regenerate kidneys by transplanting cells inside a living fetus without removing the fetal kidney (Fig. 4) [28, 29]. The blastocyst complementation method, which involves the transplantation of pluripotent stem cells into the blastocyst, remains the only method for generating chimeric organs inside the developing fetus. Chimeras that use early embryos are an ethical concern, because they may stray into non-target central nervous system cells or germ cells [30]. The use of a safer chimeric technology that utilizes cells with limited differentiation potential is one key to overcoming this concern. In an earlier study, a new method that uses progenitor cells with limited differentiation potential was developed, and in the new method, fetuses at later stages of development were used as a transplantation site [28]. In the exo utero method, the opaque myometrium is removed, leaving only the transparent fetal membranes, and the cells and elimination agent are delivered from outside the uterus to the fetal kidney using a glass tube when the inside of the uterus is easily visible [31]. NPCs are injected into the fetal kidney, and donor cells are shown to differentiate into nephrons in the host fetus (Fig. 4) [28]. Furthermore, using the aforementioned Six2-iDTR mouse fetus model of progenitor cell removal, progenitor cells were replaced inside the fetus and regeneration of the newborn nephron was shown to be connected to the host fetus [28]. This technique has not been shown to sustain the life of the fetus after birth due to difficulty with transplantation of a sufficient amount and range of cells. It is, therefore, necessary to improve the technique to allow for transplantation of sufficient amounts of NPCs in the future. Furthermore, regarding transplantation into wild-type mice, because the host renal tissue remains, the host mouse stays viable after birth even though it has a mosaic nephron, and the chimeric mouse can be raised for a long time by leaving it with its foster mother. Since regenerated tissues can be matured and maintained for long periods, it is believed that this method can be used to verify the differentiation potential of induced renal progenitor cells in vivo. There are two methods for the transplantation of renal progenitor cells into the renal niche, namely the ex vivo method, which uses PANDA, and the all-in vivo method, which involves the exo utero method. The former method has the advantage of autologous vasculature. The advantage of the latter method is that the entire developmental environment can be in utero; in addition, a second surgery for urinary tract formation is not required.

## Challenges and prospects of chimera technology

It is necessary that chimeric organs derived from two cell populations share signals in the developmental stage. However, if the adhesion factors and receptors in the host developmental environment are different, sharing of developmental signals will be difficult. Chimerism can occur between closely related species, such as rats and mice, without special intervention. However, as the distance between species increases, such as between humans and mice, the possibility of chimerism decreases [32]. In other words, the larger the gap created by interspecies differences, the less the possibility of chimerism [33]. For example, human leukemia inhibitory factor (LIF) can be used to maintain mouse embryonic stem (ES) cells, but mouse LIF has no effect on human ES cells because mouse LIF cannot bind to the human receptor [34]. Hence, due to interspecies gaps, creation of interspecies chimeras is not easy. However, if interspecies gaps can be identified and corrected, the interspecies barrier may be overcome. Chimera research with focus on immunology is ongoing. It was reported in a previous study that, to bridge the gap between species, immunologically humanized mice were created by knocking into the mouse locus each of the four human genes encoding cytokines important for innate immune cell development [35]. By further injecting human hepatocytes into these immunologically humanized mice, the livers of the mice were also humanized, and the generation of human erythrocytes circulating in mice with humanized livers and immune mechanisms was demonstrated [36]. In other words, if the discrepancy between human and animal developmental regulatory programs can be corrected, human cell differentiation/development can be supported in the host animal. While systemic chimeras derived by pluripotent stem cell integration must even out the gap between the two species from early developmental stages, it is believed that local chimeras integrating progenitor cells and late developing fetuses can focus interspecies comparisons only on the nephrogenic stage. It is desirable to construct a humanized host specialized for renal regeneration by exposing the interspecies gap that serves as a rate-limiting point for chimera formation limited to the nephrogenic stage and by appropriately modifying it using genome editing.

In experiments that focused on chimeric integration only at the nephrogenic stage, chimeric kidney tissues with interspecies connection were obtained [22]. In previous studies, it was reported that transcription factors involved in organogenesis are highly homologous and similar between different species [37]. Human and mouse chimeric kidneys were constructed in an earlier study [22], but it is still difficult to generate such kidneys using systemic chimera technology. In other words, the combination of local chimera technology using progenitor cells limited to nephrogenesis, which was originally thought to be similar to humanized host generation, and empty niche production technology (progenitor cell replacement) may be a way to overcome the barrier between distal species.

## Conclusion

From a global perspective, RRT for patients with ESKD is insufficient. The development of new RRTs should broaden the range of treatment options for ESKD and help reduce the mismatch between supply and demand. One such RRT involves the regeneration of organs from autologous stem cells to replace failing organs, which will truly be the ultimate therapy. However, it is still difficult to fully reproduce the organ architecture and differentiation in vitro. With the discovery of iPS cells as a cell source for organ regeneration, advances in analytical technologies such as omics, and improvements in genome editing technology, technology has made great strides over the last 15 years [38]. Given the progress in the field of kidney regeneration, which requires regeneration of complex organ regeneration including urological tissues in vivo, I expect that hybrid organ regeneration technology based on chimera technology that borrows the developmental environment and structures of different animal species will facilitate the realization of 3D organ regeneration.
